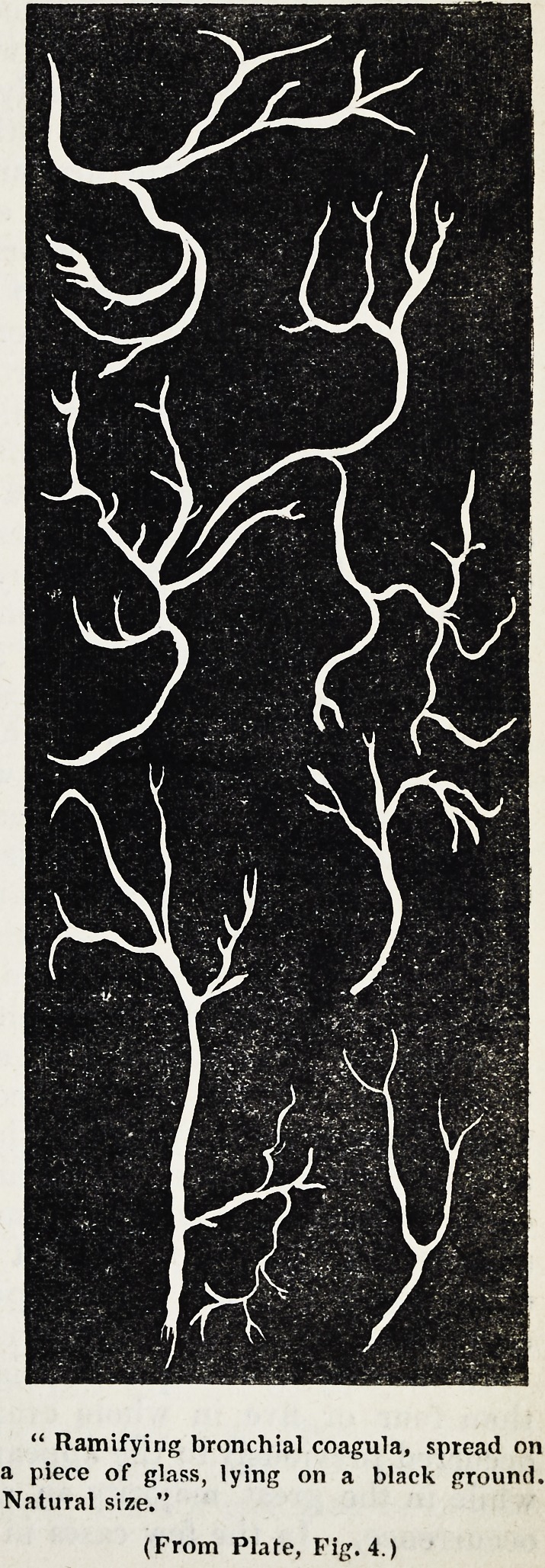# Remak's Diagnostic and Pathogenetic Investigations

**Published:** 1847-04

**Authors:** 


### ART. XIV.

*Diagnostische und Pathogenetische Untersuchungen in der Klinik des Herrn Geh. Raths Dr. Schönlein, auf dessen Veranlassung angestellt, und mit Benutzung anderweiliger Beobachtungen, veröffentlicht von* Dr. R. REMAK. Mit einer Kupfertafel.—*Berlin,* 1845.

*Diagnostic and Pathogenetic Investigations in Dr. Schönlein's Clinical Works.* By Dr. R. REMAK. With a Copper-plate.—*Berlin,* 1845. 8vo, pp. 250.

THE contents of this volume of observations are arranged chronologically, and are the results of his labours in Schönlein's *clinique* from the commencement of the winter session, 1843, to the end of the winter session, 1844. The three sessions occurring in this interval are represented by three separate departments: the first session yielding materials for eight articles, the second (the summer session of 1844) for three, and the third for ten. In consequence of this arrangement, we find observations on the same disease scattered through several parts of the book; thus we have three articles on abdominal typhus, and an equal number on pneumonia; and two on Bright's disease, glanders in the human subject, and on the signification and importance of the buffy coat of the blood. In addition to them, we have single articles on dysentery, scarlatina, spermatorrhœa, tubercle, the metamorphosis of thrombus, pus and mucus, porrigo lupinosa, fungi occurring in the mouth and intestinal canal, and certain forms of parasitic acari. From this ample store we shall endeavour to select the most important and practical matter.

*Abdominal typhus.* Remak has minutely described the microscopic characters of the excrements in this disease. After noticing the circumstance that the occurrence of crystals of ammoniaco-magnesian phosphate is not diagnostic of typhus, as Schönlein originally (Müller's Archiv, 1836) believed, he states that he failed in detecting any fixed relations between their quantity and the stage of the disease. They were generally the most frequent in the most liquid stools, and were rarest when numerous infusoria were present.

Granular cells resembling (but more delicate than) pus-corpuscles are frequently observable in the liquid evacuations. The whitish flocculi which we commonly notice in typhus evacuations, are merely fragments of undigested vegetable food. It is singular that cylinder-epithelium is never to be detected; in all probability the detached portions are altogether broken up within the intestinal canal. A small number of blood-corpuscles, more or less altered in form, may be often detected by the microscope; and fat-globules are by no means uncommon, even in cases in which no fatty matters have been administered. In one instance, on examining the diseased portion of the intestine, the epithelium investing it had a whitish appearance, arising from its cells being filled with minute dark (fat?) corpuscles.

The cryptogamic plants and the infusoria occurring in these cases are of comparatively little importance, and must be merely regarded as signs of the fermentation and putrefaction going on within the intestinal canal, in consequence of the deranged condition of the digestive functions.

*Dysentery.* Similar as are the ordinary appearances of the fæcal evacuations in dysentery and typhus, yet, examined under the microscope, they occasionally present striking differences. In the former, crystals of ammoniaco-magnesian phosphate are very rare, and the blood-corpuscles are not so much decoloured, nor so modified in form: moreover, long strings of coagulated fibrin are almost always intermingled with the corpuscles, indicating that the blood is derived from larger vessels and deeper structures. The granular cells already mentioned are, in this case, mixed with numerous flattened, spherical, and cylindrical epithelial cells, and the whole are imbedded in the structureless stroma (grundlage) of the mucus. Vibriones are scarcely ever present, but confervæ, and sometimes fermentation-fungi, occur in great excess, and apparently in a direct ratio with the degree of acidity presented by the evacuation.

*Bright's disease.* Remak confirms the view maintained by Henle, that the cylinders occurring in the urine are not modified urinary canals, but simply coagula of fibrin moulded in those canals. He conceives that the granular appearance of the cylinders is due to the presence of urate of ammonia. He regards their presence as of great importance in enabling us to establish an early diagnosis of the disease, and states that by this means he has, on more than one occasion, determined the nature of the disease several weeks before the first appearance of albumen in the urine.

*Scarlatina.* In cases of scarlatina the urine often continues to deposit a white flocculent sediment for a considerable time after the process of external desquamation has ceased. This deposit consists, for the most part, of epithelial scales from the surface of the bladder; and as long as it continues to occur, the patient must be carefully watched, even though in all other respects his health be completely re-established.

*Pneumonia.* Remak has, as we have already mentioned, contributed three articles on this subject, extending collectively over the space of thirty-four pages. After noticing how few important facts have yet been elicited in the examination of the expectoration in inflammatory affections of the air-passages, he proceeds to remark that he is fully convinced that there exists a microscopic distinction between true pus-corpuscles and the mucus-corpuscles which occur in the expectoration in cases of pulmonary inflammation. The latter differ from the former, not merely in possessing the property of absorbing water, and consequently, of becoming swollen, but likewise in the circumstance that the substance in which the nucleus is imbedded, is a fine granular, almost pulverulent matter, in which, after saturation, molecular motions may be observed. It is only after imbibition that the burst and compressed mucus-corpuscles bear any resemblance to pus-corpuscles; and even in the purulent (?) expectoration occurring in the last stages of pulmonary expectoration, Remak has never detected cells, which, in the character of their granular contents, altogether correspond with pus-corpuscles from abscesses. Moreover, the granular cells occurring in puriform sputa are always deposited in a tenacious stroma peculiar to mucus, and never present in genuine pus.

In pneumonic expectoration he not unfrequently met with dark granular, roundish bodies, considerably larger than mucus-corpuscles, and probably identical with Gluge's inflammatory globule. As he has also noticed them in the tough mucus, loosened from the fauces on clearing the throat, and likewise in the pulmonary vesicles of perfectly healthy cattle, it is difficult to decide their exact pathological importance. By far the most important of his observations in relation to the sputa in pneumonia is, that they invariably contain *ramifying bronchial coagula.* This peculiar form of expectoration may occur at different periods of the disease and in different quantities. It is not always easy of detection. Remak advises that the whole of the expectoration should be poured into a dark-coloured flat vessel filled with water, the colour enabling us to distinguish the white coagula from the mucous and puriform matter with which they are associated; or that the individual clots in which the presence of these coagula may be suspected, should be examined on a dark glass plate.

The bronchial coagula form ramifying cylinders pursuing a nearly rectilinear course, presenting a dichotomic arrangement, and gradually diminishing in length and thickness. The main stem is, however, usually thinner than the contiguous branches, and tapers off in a thread-like form at its free extremity. At the points of bifurcation we not unfrequently observe a slight dilatation, depending probably on a similar condition of the bronchial ramification. These cylindrical coagula are sometimes partially flattened, and are sometimes swollen at various points, the latter phenomenon being caused by inclosed air-bubbles.

The annexed engraving copied from Remak's treatise represents the natural size of different forms of these coagula.

" Ramifying bronchial coagula, spread on a piece of glass, lying on a black ground. Natural size."

(From Plate, Fig. 4.)

These bronchial coagula are formed of extremely delicate threads running parallel to, and closely connected with, one another, and in most cases, either inclose or are covered with granular cells, closely resembling pus-corpuscles. Their strength and mode of arrangement are suggestive of areolar tissue, but the difference becomes marked on the addition of acetic acid; for although the fibres become perfectly transparent and a number of nuclei remain, yet they are evidently the nuclei of dissolved granular cells, and altogether different from the elongated variety occurring in areolar tissue. Dr. Heintz has shown by a series of chemical experiments that these coagula are formed of a protein-compound, but whether that protein-compound is fibrin or whether it contains oxyprotein is uncertain.

The bronchial coagula appear, in the majority of cases, between the third and seventh day of the disease, being rarely absent on the fourth and fifth. This observation applies, however, only to those cases in which the proper remedies have been applied from the commencement; for in a man in whom the disease was allowed to go on unchecked, and there were signs of hepatization before any treatment was adopted, they were not apparent till the fourteenth day.

The characters of the expectoration in the three stages of pneumonia are well known: in the first stage the sputa consists of gray, stringy, viscid mucus, usually tinged with blood; in the second stage it consists of whitish, firm, clotted masses, whose white delicate fibres extend to the bottom of the vessel; and lastly, it forms soft roundish masses of a white or yellow (puriform) colour, without dependent mucus-fibrils. The bronchial coagula occur in the first and second but not in the third form. When they are found during the first stage they are very thin, not thicker than fine threads, and give off few branches; when present in the second stage they are stronger and ramify to a greater extent.

In making these examinations, the difference between the dependent white mucus-fibrils and the bronchial coagula may be easily discerned, even without the use of the microscope, by spreading the expectoration on a glass plate, when the white mucus-fibrils can be readily drawn out with a needle in the form of a delicate membrane, while the bronchial coagula, from their firmness, resist such an attempt, and are further rendered sufficiently conspicuous by their constant ramifications.

Remak next considers the connexion between the expectoration of these coagula, and the phenomena of auscultation and percussion.

The appearance of the delicate coagula imbedded in viscid mucus is generally simultaneous with that condition of the lungs in which the crepitation is the most marked while at the same time the sound evolved on percussion indicates that the substance of the lung is partially impervious to air; the firmer coagula usually occur at the period when the crepitation has given place to bronchial respiration, and the dullness on percussion indicates the existence of hepatization.

One important practical result deduced from Remak's observations, is that the earlier the expectoration of the coagula commences, and the more abundant and continuous it is, so much the more certain and speedy will be the cure. In the ordinary course of pneumonia in vigorous persons, the delicate coagula appear in the first viscid mucus that is expectorated, and continue increasing in quantity and size till the fifth day of the disease; decreasing gradually from that period, until white, easily compressible masses of a cylindrical but non-ramifying form appear in their place, exhibiting, under the microscope, indications of the fibrous structure of coagulated fibrin, and a multitude of granular mucus-corpuscles.

In the fifty cases of pneumonia observed by Remak, there were not more than four or five in whom even a partial diminution of the symptoms occurred previously to the appearance of the coagula in the expectoration, while in the great majority an amendment was first observed after their occurrence. In the few cases in which the proper remedies had not been applied as early as they ought, the appearance of the firm, ramifying, bronchial coagula, was preceded by that of soft, cylindrical, white fibrous masses, which likewise assume the form of the bronchi, but usually follow the discharge of the firm coagula, and appear to indicate their softening. Schönlein is of opinion that in these cases there is a want of sufficient energy in the lungs to eject the bronchial coagula at the due time, and that consequently, they soften in the bronchi.

Although these coagula were detected by Remak in every case of pneumonia, yet in four cases of genuine bronchitis that fell under his observation, he could not discover the slightest trace of them, but simply granular cells in a mass of ropy mucus.

Schönlein regards the presence of the bronchial coagula in the expectoration as a certain evidence that exudation is going on, in which case, unless any violent reaction should call for its adoption, general blood-letting is replaced by cupping, mercurial and iodine frictions, calomel, and diuretic and cooling medicines, such as infusion of digitalis with nitrate of potash.

*Glanders.* Remak describes two cases of acute glanders occurring in the human subject, and states that Froriep usually sees several such cases annually. After giving a description of the microscopic appearances of the lardaceous, purulent masses met with in the lungs, muscles, &c., he mentions that in relation to the diagnosis of typhus fever, it is always of the greatest importance to ascertain whether the patient has exposed himself to the possible infection of glanders, and quotes the following case:

"During the past session a waggoner aged thirty years entered the *Clinique,* having for the six previous days experienced shiverings, great depression, and pain in the muscles of the calf, thigh, and arm, on those parts being touched. The fever was moderate (pulse 90), the tongue moist and not coated; no appetite. Neither the urine nor the skin gave indication that the disease was of a rheumatic nature. From that day the pain disappeared from the affected muscles, but was felt in the left calf, in which a slightly red tumour, painful on pressure, began to form. The patient confessed that he had been engaged with diseased horses. On the third day there was pain in the muscles of the back, and in the region of the kidney, on pressure; blood-corpuscles and albumen were found in the urine, and continued to present themselves for several days. On the supposition that the disease was connected with the infection of glanders, Schönlein adopted Andral's plan of treatment with iodide of potassium, giving a scruple daily. The muscular pains, hæmaturia, and tumour then gradually disappeared. In the course of fourteen days he was altogether free from fever, but the affected muscles continued weak for a considerable time." (pp. 191-2.)

The disease in the two acute cases proved fatal on the second and fourth days respectively.

*The buffy coat of the blood.* A memoir of nearly forty pages on the signification of the buffy coat, and on the formation and production of the blood-corpuscles, terminates with the following practical remarks:

1. In order to arrive at a certain appreciation of the diagnostic and prognostic value of the buffy coat, it is requisite that the blood in all cases of venesection, should be collected in narrow and high vessels. It frequently happens that when broad vessels are used, and the coagulation proceeds irregularly, no buffy coat is formed, when if collected in another manner it would have undoubtedly appeared—a fact which, although generally known, is very little attended to in a practical point of view. Since attention has been paid in Schönlein's *Clinique* to the mode of collecting the blood, and its coagulation has been carefully watched, a buffy coat, often of considerable extent, has been observed in every case. Although the mode of bleeding pursued by Schönlein in inflammatory cases, can only be adopted when the inflammation is of a high degree, yet in some cases of pneumo-typhus and in a case of glanders, there was an extensive buffy coat containing an extraordinary number of colourless blood-cells. As it is practically impossible to make an accurate quantitative analysis of the blood in every case of venesection, we should not neglect so simple a means of arriving at an approximate determination of the amount of fibrin.

2. The microscopic examination of the buffy coat in relation to the quantity of colourless blood-cells, may be made highly useful in determining its importance as indicative of the stage of inflammation. The absence of many colourless blood-cells in the buffy coat, affords a much more certain indication of an unusually large amount of fibrin arising from inflammation, than does the presence of a great number; the latter condition being generally dependent on the regeneration of blood after repeated venesection, and probably connected with an imperfect metamorphosis of the cellular elements of the blood in discrasic diseases, such as typhus, glanders, scurvy, and cancer.

*Spermatorrhœa.* From a long and interesting memoir on the subject drawn up from the observation of forty-five cases, we select the two following passages:

"Without the confession of the patient, the occurrence of a cartilaginous hardness of the corpora cavernosa affords diagnostic evidence of long-practised habits of onanism." (p. 153, note.)

"Gonorrhœa undoubtedly predisposes to spermatorrhœa, as was shown by Lallemand. But whether it be the gonorrhœa itself, or the balsam of copaiva taken to cure it, that causes this predisposition, it is difficult to say. I regard the latter as probable, since it has been noticed by Schönlein to induce baneful effects on many persons who have taken it for gonorrhœa in full doses, and for a long time, giving rise to Bright's disease." (pp. 168-9.)

*Porrigo lupinosa.* Remak carefully examined the scabs in a large number of cases of porrigo lupinosa, in order to ascertain whether the fungous structures contained in them were always identical. His observations on this point coincide with those instituted by Schönlein at Zürich, Fuch's and Langenbeck at Göttingen, Gruby at Paris, and Bennett at Edinburgh, and thus tend to show that geographical position exerts no influence on the species of the vegetable parasite.

A hair is usually found in the centre of each scab, and around the hair there are concentric furrows, dividing the scab into a number of rings averaging the fourth of a line in breadth. The scab increases by the augmentation of the external ring.

On cutting through one of the older scabs, after its removal from the body, we observe two distinct strata separated by a boundary line; the one next to the skin being thin, whitish and brittle, while the other is thick and yellow, forming the free surface of the scab. In the white layer is the thallus, while in the yellow the filaments (Sporidienträger) and sporidia predominate. After a copious description of the microscopic characters of these minute fungi, he proceeds to consider the place they ought to hold amongst the cryptogamia. From the examination of specimens sent by him to G. R. Link and Dr. Klotzsch, those botanists are of opinion that the favus-fungus is a distant species, and not to be classed under either torula or oidium. The latter rather inclines to regard it as a species of the genus sporotrichum. Remak proposes for it the name of achorion Schönleini, derived from achor, the old name for favus; and gives the following botanical characters of the plant:

"Achorion Schönleini nobis, orbiculare, flavum, coriaceum, cuti humanæ præsertim capitis insidens; rhizopodion molle, pellucidum, floccosum, floccis tenuissimis, vix articulatis, ramosissimis, anastomoticis (?); mycelium floccis crassioribus, subramosis, distincte articulatis, articulis inæqualibus irregularibus in sporidia abeuntibus; sporidia rotunda, ovalia vel irregularia, in uno vel pluribus lateribus germinantia."

He then proceeds to relate a series of experiments on the distribution and growth of these fungi, and gives a circumstantial account of a successful attempt at inoculating his own arm. The mode of formation of the scab, and the chemical character of fluids conducing to the development of the fungi are then considered.

We shall terminate our notice of this section with a quotation containing a sound practical truth—one that should never be lost sight of in the treatment of this class of diseases:

"Eruptions on the heads of children appear to take the place of other morbid processes in more important organs; they are found to alternate with chronic inflammation and mucous discharges of the conjunctiva and cornea, and of the external auditory meatus, with enlargement and suppuration of the cervical glands, with enlargement and atrophy of the mesenteric glands, and probably also with tubercles of the lungs, bones, and intestinal canal. . . . It is very possible that the irritation and suppuration of the skin, produced by the growth of the fungous scabs, may in certain cases exercise a favorable influence on the condition of the organism." (p. 213.)

*Fungi in the mouth and intestinal canal.* In his observations on the microscopic characters of the aphthæ of children, and of adults (during typhus), Remak found that a single aphtha often contained several species of fungi, and that the species of fungi occurring in different aphthæ on the same individual exhibited no constant relationship; also that while one aphtha contained numerous fungi, others on the same individual contained none at all. Hence it appears that the loosening (auflockerung) and ulceration of the mucous membrane is the primary phenomenon, and that the fungi are only produced under favorable chemical conditions. It has been long known that the presence of aphthæ is usually associated with an excess of acidity in the primæ viæ, and it is very probable that a putrid condition of the surface of the mucous membrane would accelerate the conversion of sugar and starch into lactic acid, and thus afford a favorable soil for the growth of these fungi.

Even in health there are produced during sleep a quantity of confervæ in the cavity of the mouth, which adhere to the mucous membrane and the teeth. They are probably occasioned by the decomposition of particles of food, or of mucus. In a young man suffering from hoarseness, Remak found that the mucus which was secreted in great quantity by the soft palate, contained a large number of ramifying thallus-filaments. In diseases of the air-passages in which the epithelium of the mouth and throat is being frequently abraded and renewed, traces of cryptogamic plants may be generally detected in the expectoration. In the bronchial coagula of pneumonia patients (vide supra p. 505), Remak constantly found a peculiar variety of bifurcating, ramifying thallus-filaments, whose length and degree of development indicated that they could not have formed subsequently to the expectoration of the coagula.

There is a remarkable difference between the pathogenetic relations of the achorion and the fungi growing in the cavity of the mouth, and probably also in aphthæ. The achorion takes deep root in the sound uninjured cutis, which indeed seems to be the only place where it attains its full development. The fungi of the mouth, throat, and air-passages are, on the other hand, merely secondary products, formed from the decomposition of the mucous membrane and of foreign substances (fragments of food, &c.) in contact with it, and are analogous to fungi which, under proper chemical conditions, become developed out of the body. The achorion may be just as correctly termed a parasitic plant as the *acarus scabiei* or *a. folliculorum* may be termed parasitic animals; but these fungi can no more be regarded as true parasites any more than the infusoria which are abundantly present in the fluid putrid evacuations from the bowels in typhus.

A microscopic examination of the brown, chocolate-like masses observed in the sour vomited matters in cases of cancer of the stomach, has led to the interesting discovery that they consist for the most part of yeast-plants. In one case the fermentation was accompanied by a well-marked development of gas, and there was an obvious separation of the yeast into an upper and lower stratum.

We have endeavoured in the preceding pages to lay before our readers an abstract of the most important facts and observations contained in Remak's volume, and it only remains for us to express our earnest hope that this is only the commencement of a long series of our author's "diagnostic and pathogenetic investigations."

---

## Figures and Tables

**Fig. 4. f1:**